# Secondary basophilic leukemia in Ph-negative myeloid neoplasms: A distinct subset with poor prognosis

**DOI:** 10.1016/j.neo.2021.09.010

**Published:** 2021-10-31

**Authors:** Daniela Berger, Karin Bauer, Christoph Kornauth, Susanne Gamperl, Gabriele Stefanzl, Dubravka Smiljkovic, Christian Sillaber, Peter Bettelheim, Paul Knöbl, Ana-Iris Schiefer, Georg Greiner, Renate Thalhammer, Gregor Hoermann, Ilse Schwarzinger, Philipp B. Staber, Wolfgang R. Sperr, Peter Valent

**Affiliations:** a Department of Internal Medicine I, Division of Hematology and Hemostaseology, Medical University of Vienna, Vienna, Austria; b Ludwig Boltzmann Institute for Hematology and Oncology (LBI HO), Medical University of Vienna, Vienna, Austria; c Department of Pathology, Medical University of Vienna, Vienna, Austria; d Division of Hematology and Oncology, Elisabethinen Hospital Linz and Europa-Platz Labor Linz, Linz, Austria; e Ihr Labor, Medical Diagnostic Laboratories, Vienna, Austria; f Department of Laboratory Medicine, Medical University of Vienna, Vienna, Austria; g Munich Leukemia Laboratory (MLL), Munich, Germany

**Keywords:** Basophils, Basophilic leukemia, IgE receptor, Azacitidine, Venetoclax

## Abstract

During progression of myeloid neoplasms, the basophil compartment may expand substantially and in some of these patients, a basophilic leukemia is diagnosed. In patients with Ph-chromosome+ chronic myeloid leukemia, acceleration of disease is typically accompanied by marked basophilia. In other myeloid neoplasms, secondary leukemic expansion of basophils is rarely seen. We report on 5 patients who suffered from a myelodysplastic syndrome, myeloproliferative neoplasm, or acute leukemia and developed a massive expansion of basophils during disease progression. In 4 of 5 patients, peripheral blood basophil counts reached 40%, and the diagnosis “secondary basophilic leukemia” was established. As assessed by flow cytometry, neoplastic basophils expressed CD9, CD18, CD25, CD33, CD63, PD-L1, CD123, and CLL-1. In addition, basophils were found to display BB1 (basogranulin), 2D7, tryptase and KIT. In 4 of 5 patients the disease progressed quickly and treatment with azacitidine was started. However, azacitidine did not induce major clinical responses, and all patients died from progressive disease within 3 Y. In *in vitro* experiments, the patients´ cells and the basophilic leukemia cell line KU812 showed variable responses to targeted drugs, including azacitidine, venetoclax, hydroxyurea, and cytarabine. A combination of venetoclax and azacitidine induced cooperative antineoplastic effects in these cells. Together, secondary basophilic leukemia has a poor prognosis and monotherapy with azacitidine is not sufficient to keep the disease under control for longer time-periods. Whether drug combination, such as venetoclax+azacitidine, can induce better outcomes in these patients remains to be determined in future clinical studies.

## Introduction

Persistent peripheral blood (PB) basophilia is typically seen in patients with myeloid neoplasms, especially when the disease progresses [[1], [2], [3], [4], [5], [6], [7], [8], [9]]. For example, in patients with Ph-chromosome+ chronic myeloid leukemia (CML), basophilia is a typical finding, and the numbers of basophils increase further with acceleration of the disease [1−4,9]. Basophilic leukemia is a rare myeloid neoplasm characterized by an excessive, uncontrolled expansion of basophils. As per definition, basophils account for 40% of all leukocytes in these cases [8]. Basophilic leukemia can be divided into acute and chronic forms (based on the blast cell count) and into primary variants (no known preceding myeloid neoplasm) and secondary leukemia [8]. Secondary basophilic leukemia is most commonly diagnosed in patients with CML and is defined by the Philadelphia (Ph) chromosome [2,7−9]. Other forms of secondary basophilic leukemia have also been described, but are exceptionally rare [5,6,8]. In some of these patients, an underlying myelodysplastic syndrome (MDS), myeloproliferative neoplasm (MPN) or MDS/MPN overlap neoplasm is diagnosed [5,6]. The prognosis in all these variants of basophilic leukemia is grave, independent of the primary neoplasm.

Treatment of secondary basophilic leukemia has to be adjusted to the underlying myeloid neoplasm, the age of the patient, and the availability of a stem cell transplant donor [8]. In those, who have an underlying CML, treatment with second and/or third-line BCR-ABL1 tyrosine kinase inhibitors (TKI), poly-chemotherapy, and/or hematopoietic stem cell transplantation (HSCT) have to be considered. Similarly, in patients with MDS and acute myeloid leukemia (AML), poly-chemotherapy and HSCT are often recommended, provided that the patient is eligible. However, not all patients can be treated with poly-chemotherapy and HSCT or they refuse such intensive treatment. In these patients, alternative drugs have to be offered.

One reasonable “conventional” treatment approach for such cases may be the use of hypomethylating agents, such as azacitidine or decitabine, or low-dose cytarabine [10,11]. Another potential drug candidate is venetoclax, a BCL-2-targeting agent that has been used successfully in patients with high-risk or chemotherapy-resistant MDS or AML [12,13]. We report on 5 patients with advanced myeloid neoplasms and substantial basophilia. In 4 patients, the diagnosis of secondary basophilic leukemia could be established. Three patients received azacitidine. However, responses were only transient and followed by a relapse. All patients died within 3 Y.

## Patients and methods

### Patients

PB and/or bone marrow (BM) was obtained during routine investigations (diagnostic- or follow-up samples) from 5 patients with myeloid neoplasm, namely 1 with MDS, 2 with AML, 1 with MDS/MPN, and 1 with MPN (primary myelofibrosis). Diagnoses were established by using criteria provided by the World Health Organization (WHO) [14]. The patients’ characteristics are shown in Table 1. Routine investigations included PB counts and differential counts, morphologic examination of cells on BM smears, BM histology and immunohistochemistry (IHC), analysis of BM cells for cytogenetic abnormalities, and molecular aberration profiling by next generation sequencing (NGS). Conventional karyotyping and fluorescence in situ hybridization were performed according to standard methodologies [15]. The study was approved by the local ethics committee of the Medical University of Vienna. Informed consent was obtained from all patients before PB and/or BM was obtained. Isolated mononuclear cells (MNC) were stored in a local biobank.

**Table 1:** Patients´ characteristics.

No #	Age (Y)	F/M	Diagnosis	WBC (G/L)	PB BA (%)	BM BA (%)	Splenomegaly Yes/No	Therapy	Best Response	Survival (mo)
1	67	M	MDS-ba;RC-MLD-baCBL	9.42	61	14	yes	Azacitidine	Stable disease	3
2	77	M	sAML-ba	8.7	31	04	no	Azacitidine	partial hematologic response^a^	13
								Venetoclax	Blast cell reduction	1.5
3	65	M	AML; ABL	34.2	40	35	yes	3+7	No response	
								FLAG+DA	No response	6
								SCT	Early relapse	2
4	75	F	PMF-ba; ABL	15.9	43	n.d.	yes	Azacitidine + Hydroxyurea	No response	2
5	84	F	MDS/MPN-baCBL	41.4	48	05	no	Hydroxyurea	Stable disease	32

Clinical features of patients at the time of basophil crisis, therapy, response to therapy and survival of patients are shown.

ABL = acute basophilic leukemia; AML = acute myeloid leukemia; ba = basophils, BM = bone marrow; CBL = chronic basophilic leukemia; F = female; FLAG+DA = chemotherapy with fludarabine, cytarabine, and G-CSF + dasatinib; m = male; MDS = myelodysplastic syndrome; MPN = myeloproliferative neoplasm; n.d. = not determined; PB = peripheral blood; PMF = primary myelofibrosis; RC-MLD = refractory cytopenia with multilineage dysplasia; SCT = stem cell transplantation; WBC = white blood count.

a The patient initially received azacitidine as monotherapy and entered a good hematologic response with complete peripheral blast cell clearance (<1%) within 4 mo (no further bone marrow investigations were performed during therapy). Because of persisting severe pancytopenia, he also received granulocyte colony-stimulating factor (G-CSF) and romiplostim which resulted in an increase in platelet counts (>100,000/µL) and neutrophils (>neutrophils/µL). After 1 Y, however, blast cells again increased (>75%) and severe thrombocytopenia was again noted. The patient then received venetoclax which resulted in a blast cell reduction, but without complete remission. The patient died after 1.5 mo of therapy with venetoclax.

### Drugs and other reagents

Ponatinib, cladribine, cytarabine, selinexor, JQ1 and venetoclax were purchased from Selleck Chemicals (Houston, TX, USA), dasatinib from ChemieTek (Indianapolis, IN, USA), rapamycin from Calbiochem (San Diego, CA), hydroxyurea (HU) and azacitidine from Sigma-Aldrich (St. Luis, MO, USA). Stock solutions of most drugs were prepared by dissolving in dimethylsulfoxide (DMSO; Sigma-Aldrich); azacitidine was dissolved in 50% acetic acid, and HU in distilled water. RPMI 1640 medium and antibiotics (penicillin, streptomycin) were purchased from Lonza (Basel, Switzerland), amphotericin B from PAN‐Biotech (Aidenbach, Germany), fetal calf serum (FCS) from Gibco Life Technologies (Carlsbad, California, USA), and ^3^H-thymidine from Perkin Elmer (Waltham, MA, USA).

### Culture conditions for primary cells and KU812 cells

Primary cells were obtained from the patients´ PB or BM samples and stored in a local biobank. In 1 patient, splenectomy was performed (for cytoreduction and to improve cytopenia) and splenic cells were collected and stored. MNC were isolated by Ficoll density gradient centrifugation and used in immunocytochemistry (ICC) staining experiments, flow cytometry, and drug incubation experiments. The percentage of basophils in the MNC fractions ranged between 8% and 48% by Wright-Giemsa staining. For histamine release experiments, basophils were enriched by dextran sedimentation as reported [16]. The BCR-ABL1+ basophilic cell line KU812 [17] was kindly provided by Dr. Kenji Kishi (Niigata University, Niigata, Japan). Cells were cultured in RPMI 1640 medium supplemented with 10% FCS and antibiotics in 5% CO2 at 37°C.

### IHC and ICC

IHC was performed on slides prepared from paraffin-embedded, formalin-fixed BM biopsy specimens using the indirect immunoperoxidase staining technique following established protocols [18,19]. Endogenous peroxidase was blocked by methanol/H_2_O_2_, followed by heat-induced or protease-induced epitope retrieval. Mouse monoclonal antibodies (mAb) against human tryptase (1:100), chymase (1:100), basogranulin (1:50), 2D7 (1:25), and CD30 (1:50), and a rabbit polyclonal antibody against CD117 (1:100) were applied at 4°C overnight. A specification of antibodies is provided in Supplemental Table S1. Biotinylated goat anti-rabbit or goat anti-mouse IgG (Vector Laboratories, Burlingham, CA, USA) were applied as secondary antibodies (work dilution 1:200) for 30 min, washed, and then exposed to Vectastain ABC KIT (Vector) for 30 min. 3-amino-9-ethylcarabzole (AEC; Sigma-Aldrich) was used as chromogen substrate. Finally, slides were counterstained in Mayer´s hematoxylin (Morphisto, Frankfurt am Main, Germany). ICC was performed using primary MNC as described [18,19]. Cells were spun on cytospin slides and fixed with acetone. Slides were incubated with antibodies against tryptase (1:100), chymase (1:50), basogranulin (1:100), 2D7 (1:100), and CD30 (1:50); and a rabbit polyclonal antibody against CD117 (1:100) at 4°C overnight. A specification of antibodies is provided in Supplemental Table S1. Slides were incubated with biotinylated goat anti-rabbit or goat anti-mouse IgG (Biocare Medical, Walnut Creek, CA, USA) for 30 min at room temperature and then with streptavidin AP label (Biocare Medical, Walnut Creek, CA, USA) for 30 min. Neofuchsin (Nichirei, Tokyo, Japan) was used as chromogen. All slides were counterstained in Mayer´s hematoxylin.

### Evaluation of drug effects on proliferation of basophils

To determine the antiproliferative effect of drugs on patient-derived MNC and KU812 cells, ^3^H-thymidine uptake experiments were performed. Primary MNC or KU812 cells were incubated in control medium or in various concentrations of HU (5−5000 µM), azacitidine (0.5−100 µM), venetoclax (0.01−10 µM), cytarabine (0.001−10 µM), cladribine (0.001−10 µM), selinexor (0.001−10 µM), JQ1 (0.001−10 µM), rapamycin (0.001−10 µM), ponatinib (0.01−10 nM), or dasatinib (0.01−10 nM) at 37°C for 48 h. To assess cooperative drug effects, KU812 cells and primary MNC were incubated in various concentrations of azacitidine, venetoclax, or HU, and in combinations of these drugs. Then, ^3^H-thymidine was added, and its uptake was analyzed after 16 h using a beta-counter (Perkin Elmer). All experiments were performed in triplicates.

### Flow cytometry analyses

CD203c^+^ basophils were subjected to phenotyping in all 5 patients. Multicolor flow cytometry was performed using fluorochrome-conjugated mAb on a FACSCanto (BD Biosciences, San José, CA, USA) essentially as decribed [20,21]. A specification of mAb used in these studies is shown in Supplemental Table S2. All staining reactions were controlled by isotype-matched control antibodies. Results are expressed as staining index (SI), calculated as a ratio of median fluorescence intensities (MFI) obtained with specific antibodies and isotype matched control antibodies. In a separate set of experiments, whole blood cells were incubated with various concentrations of a polyclonal anti-IgE antibody (0.1-5 µg/ml) (Sigma‐Aldrich) at 37°C for 15 min. Then, cells were stained with fluorochrome-labeled mAb against CD13, CD63, CD164 or CD203c (15 min, 37°C). Thereafter, cells were subjected to erythrocyte lysis and analyzed by multicolor flow cytometry on a FACSCanto. Basophils were identified as CD203c^+^ cells. Anti-IgE-induced upregulation of CD13, CD63, CD164 and CD203c on basophils was calculated from MFI obtained from stimulated (MFIstim) and unstimulated (MFIcontrol) cells and expressed as stimulation index (=MFIstim: MFIcontrol).

### Histamine release experiments

The histamine release assay was performed on dextran-enriched basophils of patients #1, #2, #3 and #4 essentially as described [16,21]. Dextran-enriched basophils (purity: #1: 45%; #2; not available; #3: 23%; #4: 3%) were incubated in histamine release buffer (Immunotech, Marseille, France) in the absence or presence of polyclonal anti-IgE antibody (0.01−10 µg/ml) at 37°C for 2 h. After incubation, cells were centrifuged at 4°C, and the cell-free supernatants and total suspensions were analyzed for histamine content by radioimmunoassay (Immunotech). Histamine was measured in the supernatants and cell lysates and histamine release was expressed as percentage of total cellular histamine. All experiments were performed in triplicates.

### DNA isolation and NGS analysis

Genomic DNA (gDNA) was extracted from PB or BM cells using the QIAsymphony Sp Instrument with the QIAsymphony DNA Midi Kit (Both Qiagen, Hilden, Germany). Quantification of gDNA was performed by fluorometric quantitation using the Qubit dsDNA BR Assay Kit and a Qubit 3.0 Fluorometer (both Thermo Fisher Scientific, Waltham, USA) and diluted to a final concentration of 200 ng. Library preparation was performed with the myeloid solution panel (Sophia Genetics, Lausanne, Switzerland) according to the manufacturer's instructions. MiSeq System (Illumina, San Diego, California, USA) was used as sequencing platform. Variant calling was performed using the DDM Software (Sophia genetics).

### Statistical analysis

To define significance levels in growth inhibition experiments using targeted drugs, ANOVA with Bonferroni correction was applied. Results were considered significant when *P* was <0.05. Drug combination effects (additive vs synergistic) were determined by calculating combination index (CI) values using Calcusyn software (Calcusyn; Biosoft, Ferguson, MO, USA). A CI value of 1 indicates additive effects and a CI below 1 indicates synergistic drug interactions.

## Results

### Clinical presentation, course, response to drugs and survival

At initial presentation, patients were diagnosed to suffer from MDS (n = 1), AML (n = 2), MDS/MPN (n = 1), and primary myelofibrosis (n = 1). Peak basophil counts and the patients´ clinical features at the time of basophil leukemia or basophil crisis are shown in Table 1. In 4 of 5 patients, PB basophil counts peaked to ≥40% so that the diagnosis “secondary basophilic leukemia” was established. In 2 of these patients, secondary acute basophilic leukemia (ABL) was diagnosed, and in the 2 other patients, chronic basophilic leukemia (CBL) was diagnosed (Table 1). An overview of the clinical course and survival of our patients is shown in Table 1. In 4 of 5 patients, the disease progressed quickly. In 3 of these patients, treatment with azacitidine (with or without HU) was started. However, azacitidine did not induce major clinical responses, and all patients died from progressive disease within 3 Y. The 2 patients with secondary ABL died after 2 mo and 6 mo, respectively. One patient with MDS/MPN and secondary CBL showed a more chronic course and received HU for several months. The survival time in this patient was 32 mo (Table 1).

### Cytogenetic and molecular abnormalities detected in neoplastic cells

Cytogenetic data were obtained on BM cells in all patients. In 2 patients (#1 and #5) a normal karyotype was found. In patient #3, clonal cells were found to express a t(5;12)(q31;p13) as well as a 5q deletion. In patient #4, a deletion in 11q (*MLL*) as well as translocation t(4;14) was detected. An overview of cytogenetic abnormalities detected in our patients is provided in Supplemental Table S3. Supplemental Table S4 shows an overview of molecular abnormalities detected in neoplastic cells by NGS. In most patients, 2 or more gene abnormalities (mutations) were detected. In 1 patient (#3), 2 *TP53* mutations (exon 7: C722T and exon 5: G481A) were detected in neoplastic cells but no other genetic anomalies were identified. In 1 patient (#4) the MPN-related driver mutation *JAK2* V617F was detected. *KIT* D816V and *BCR-ABL1* were not detected in neoplastic cells by PCR or NGS in any of our patients (Supplemental Table S4).

### Expression of basophilic markers in neoplastic cells

**Fig. 1:**
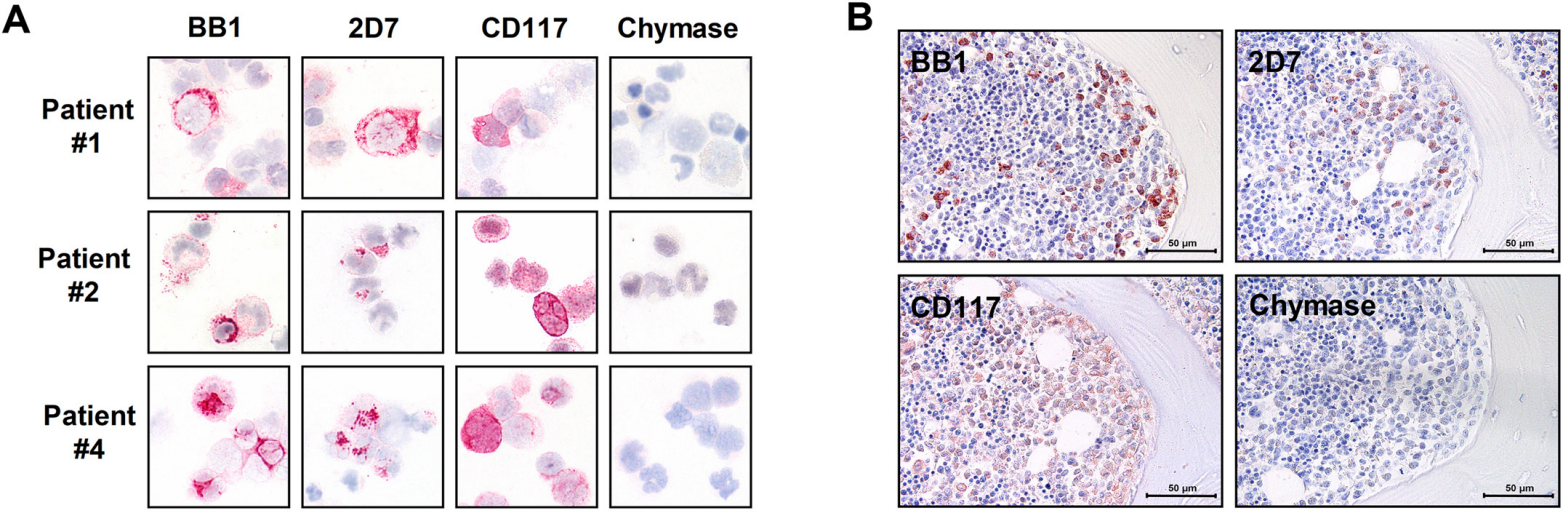
Expression of basophil markers in primary neoplastic cells. (A) Immunocytochemical detection of basophil- and/or mast cell-related antigens in neoplastic cells obtained from patients #1 (upper panel), #2 (middle panel) and #4 (lower panel). Neoplastic cells prepared on cytospin slides were stained by antibodies against BB1, 2D7, CD117 and chymase. Images were taken at a magnification of x1000. (B) Expression of basophil- and/or mast cell-related antigens in neoplastic cells was determined by immunohistochemistry on paraffin-embedded and formalin-fixed bone marrow sections in patient #1 using antibodies against BB1, 2D7, CD117 and chymase. Images were taken at a magnification of x600. Numbers of patients (#) refer to the identification numbers shown in Table 1.

As assessed by ICC, primary MNC of all patients tested (#1, #2, #4) were found to express the basophilic markers BB1 and 2D7. Furthermore, leukemic cells stained positive for CD117 and tryptase, but did not react with antibodies directed against CD30 or chymase (Fig. 1A, Table 2). In BM sections obtained from patients #1, #2 and #5, 5% to 15% of cells (mature basophils) stained positive for BB1 and 2D7. At the time of initial diagnosis, basophils were not elevated in patient #4 and most neoplastic cells failed to react with antibodies against BB1 and 2D7 (Fig. 1B, Table 2) and the same was found in control BM sections (healthy donors, not shown). Patient #2 showed a higher percentage of tryptase-positive cells (15%) and CD117-positive cells (40%) in BM sections compared to patient #1 (tryptase: 3%, CD117: 20%) or patient #4 (tryptase: 3%, CD117: <1%). Antibodies against CD30 and chymase did not react with neoplastic cells, suggesting that neoplastic cells were basophils but not mast cells.

**Table 2:** Expression of basophil-related markers in patients (#) with basophilia.

	Immunohistochemistry				Immunocytochemistry		
Antigen	#1	#2	#4	#5	#1	#2	#4
BB1	+	+	-	+	+	+	+
2D7	+	+/-	-	+	+	+	+
Tryptase	+/-	+	+/-	+/-	+/-	+	+
Chymase	-	-	-	-	+/-	-	-
CD117	+	+	-	n.t.	+	+	+
CD30	-	-	-	-	-	-	+/-

The reactivity of neoplastic cells with antibodies directed against myeloid differentiation antigens (BB1, 2D7, CD30, CD117, chymase, tryptase) in bone marrow sections (patients #1; #2; #4; #5) and on cytospin slides (patients #1; #2, #4) were determined by microscopy and are provided as percentage of total cells using the following score: +, >5%; +/-, 1-5%; -, <1%.

### Cell surface membrane phenotyping of leukemic basophils

To confirm the presence of basophils, multicolor flow cytometry experiments were performed on primary patient-derived PB or BM cells using mAb. In these experiments, CD203c^+^ basophils were found to express substantial amounts of CD11b, CD18, and CD33, and high levels of CD123, confirming that these cells are basophils (Table 3; Supplemental Figure S1). Leukemic basophils also expressed CD25, CD63, CD116, CD274 (PD-L1), and CD371 (CLL-1). In all patients examined, basophils expressed low amounts of CD117 (KIT), an antigen that is expressed abundantly in human tissue mast cells and less abundantly in basophil precursor cells. Leukemic basophils did not react with antibodies against CD2, CD30 or CD279 (PD1). In all samples tested, leukemic basophils were found to express the alpha chain of the FcεRI (IgE receptor alpha chain) (Table 3; Supplemental Figure S1).

**Table 3:** Expression of surface molecules on CD203c^+^/CD14^–^ basophils in basophilic leukemia patients (#1, #2, #3, #4) and in normal peripheral blood.

Surface Molecule	CD	#1	#2	#3	#4	#5	Normal PB
LFA-2	CD2	-	-	-	-	n.d.	-
Tetraspanin-29	CD9	+++	+++	++	++	n.d.	+++
Integrin alpha-M	CD11b	++	+	++	++	++	++
Integrin beta-2	CD18	++	+/-	++	++	n.d.	++
IL-2RA	CD25	++	+/-	+	++	n.d.	+
DPPIV	CD26	+	-	++	+/-	+/-	+
Ki-1	CD30	-	-	+/-	+/-	n.d.	-
Siglec-3	CD33	++	+	++	++	+	++
Tetraspanin-30	CD63	+	+	+	++	n.d.	++
GM-CSFR alpha	CD116	+	n.d.	+	+	n.d.	+
SCFR/KIT	CD117	+	+/-	+	+/-	-	-
IL-3RA	CD123	+++	++	+++	+++	++	+++
PD-L1	CD274	+	+/-	+	+	+/-	+
PD-1	CD279	-	-	-	-	n.d.	-
Siglec-6	CD327	+	+/-	+	+	n.d.	+/-
CLL-1	CD371	+	+	++	++	n.d.	++
FcεRI	n.c.	++	n.d.	++	++	++	n.d.
IL-1RAP	n.c.	+	+/-	+	+/-	n.d.	-

The table shows a summary of flow cytometry staining results of basophils obtained from patients #1, #2, #3, #4, and from normal PB. Staining index (SI) for CD203c+ basophils was calculated as ratio of median fluorescence intensities obtained with specific antibodies and isotype-matched control antibodies. Expression (SI) levels were scored as follows: –, SI <1.30; +/–, SI 1.31-3.00; +, SI 3.01-10.00; ++, SI 10.01-100; +++, SI >100.

CD = cluster of differentiation; CLL-1 = C-type lectin-like molecule-1; DPPIV = dipeptidyl peptidase-4; FcecL = high-affinity IgE receptor 1; GM-CSFR alpha = granulocyte-macrophage colony-stimulating factor receptor alpha; IL-1RAP = interleukin-1 receptor accessory protein; IL-2RA = interleukin-2 receptor alpha; IL-3RA = interleukin-3 receptor alpha; LFA-2 = lymphocyte function-associated 2; n.c. = not clustered; n.d. = not determined; PB = peripheral blood; PD-1 = programmed cell death protein 1; PD-L1 = programmed cell death 1 ligand 1; SCFR = stem cell growth factor receptor; Siglec-3 = sialic acid binding immunoglobulin like lectin 3.

### Effect of cross-linking of FcεRI on histamine release and expression of activation-linked antigens in leukemic basophils

In a next step, we asked whether leukemic basophils express functional IgE receptors. To address this question, we performed IgE receptor cross-linking experiments. In particular, we incubated dextran-enriched basophils of 4 patients (#1, #2, #3, #4) with various concentrations of anti-IgE (0.01-10 µg/ml) and determined histamine release. In these experiments, anti-IgE was found to induce a dose-dependent release of histamine from basophils in patients #1 and #3 (Supplemental Figure S2A). By contrast, in patients #2 and #4, anti-IgE did not induce significant histamine release from basophils (Supplemental Figure S2A). In a separate set of experiments, we also examined the effects of anti-IgE-induced cross-linking of the FcεRI on expression of activation-linked cell surface antigens on basophils. As visible in Supplemental Figure S2B, the activation-linked surface antigens CD13, CD63, CD164 and CD203c variably increased in expression on leukemic basophils after exposure to anti-IgE.

### Effects of antineoplastic drugs on proliferation of leukemic basophils

In KU812 cells, a BCR-ABL1-positive basophil-committed cell line, several drugs, including cytarabine, ponatinib and dasatinib, were found to block proliferation. Ponatinib and dasatinib suppressed the proliferation of KU812 cells with IC_50_ values ranging between 0.1 and 0.5 nM (Supplemental Table S5). By contrast, most of the other drugs, including HU, venetoclax and rapamycin, did not exert major growth-inhibitory effects in KU812 cells (Supplemental Table S5). Supplemental Figure S3 shows the effects of HU, azacitidine, venetoclax and cytarabine (ARA-C) on growth of KU812 cells. Similar results were obtained with primary neoplastic basophils in patients #2 and #4. Again, azacitidine and cytarabine were found to inhibit proliferation in primary leukemic basophils (Supplemental Table S6, Supplemental Figure S4). In addition, the BET inhibitor JQ1 and the export inhibitor selinexor showed potent antileukemic effects in these cells (Supplemental Table S6). Dasatinib and ponatinib produced varying effects on growth of primary neoplastic cells, with IC_50_ values ranging between 0.001 and >10 µM (Supplemental Table S6, Supplemental Figure S4).

### Venetoclax and azacitidine synergize in inducing growth inhibition in neoplastic basophils

**Fig. 2:**
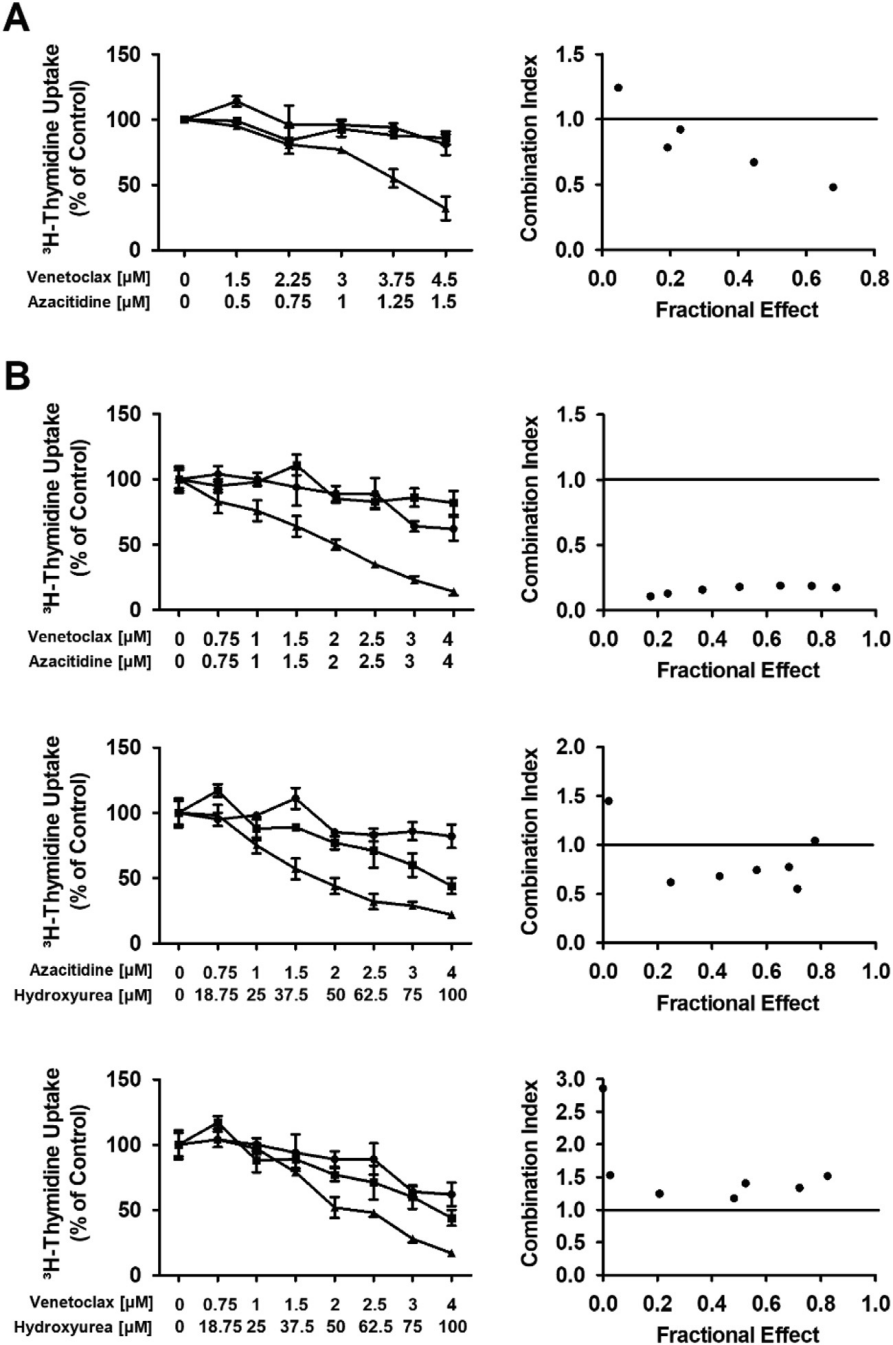
Synergistic effects of venetoclax and azacitidine on proliferation of basophils. KU812 cells (A) and leucocytes derived from patient #4 (B) were incubated in control medium (0), in medium containing various concentrations of venetoclax or azacitidine alone or in combination at a fixed ratio of drug-concentrations (as indicated) at 37°C for 48 h. After incubation, uptake of ^3^H-thymidine was measured. Results are expressed as percent of control and represent the mean±SD of triplicates (left panel). In the right panel, combination index (CI) values, calculated from fractional effects by Calcusyn software, are shown. A CI value of 1 indicates an additive effect and CI values below 1 are indicative of synergistic drug effects.

While azacitidine is an established hypomethylating drug, venetoclax acts on a specific target (BCL-2) to promote apoptosis. Thus, we hypothesized that these 2 drugs could produce cooperative antileukemic effects in leukemic basophils. To test this hypothesis, we performed ^3^H-thymidine uptake experiments and found that azacitidine and venetoclax in a ratio of 1:3 synergize with each other in producing growth inhibition in KU812 cells (Fig. 2A). No synergistic proliferation-inhibitory effects were seen when combining HU with venetoclax or HU with azacitidine in KU812 cells (Supplemental Figure S5). Next, we examined drug combination effects in primary neoplastic cells of patient #4. We found synergistic effects of the drug combination azacitidine + venetoclax (concentrations ratio, 1:1) and azacitidine + HU (concentrations ratio, 1:25) whereas no synergistic inhibition of proliferation was seen with venetoclax + HU (concentration ratio 1:25) (Fig. 2B). Synergistic drug effects were confirmed by calculating combination index values using Calcusyn software.

## Discussion

Basophilic leukemia is a rare hematopoietic neoplasm characterized by a massive increase in the number of clonal basophils in the BM and peripheral blood [5−8,[22], [23], [24], [25], [26], [27], [28], [29]]. The disease can present as an acute leukemia or as a chronic leukemia, often in the context of an underlying myeloproliferative disorder [5−8,[22], [23], [24], [25], [26], [27], [28], [29], [30], [31], [32]]. In a subset of patients, certain driver genes, such as *BCR-ABL1* or *JAK2* V617F, are expressed in neoplastic cells [8,22−32]. Depending on the underlying disease and molecular targets detected in neoplastic cells, the disease may respond to (targeted or conventional) antineoplastic drugs. Overall, treatment responses are mostly transient and the prognosis is poor [5−8,22−32]. In a subset of patients, an underlying myeloid neoplasm is detected initially, and later, basophil crisis or basophilic leukemia develops [5−8,23−25,[33], [34], [35], [36]]. We report on 5 patients with secondary Ph-negative basophilic leukemia or basophil crisis. In all patients, responses to treatment were transient and poor and all patients succumbed their disease within 2.5 Y.

In the past few decades, several proposals for the definition and classification of basophilic leukemias have been proposed [8,14,30]. While previous studies have differentiated Ph-positive from Ph-negative forms, more recent proposals and the WHO classification separates CML from basophilic leukemia [14,37]. We have recently proposed a classification in which basophilic leukemia is defined by a threshold of 40% basophils and is divided into acute (ABL) and chronic forms (CBL), based on the percentage of blast cells [8]. Using this classification, 2 of our patients were classified as ABL and 1 as CBL. In 1 patient (#2) diagnosed with secondary AML, basophil counts reached 31% but did not exceed 40%. Therefore, although closely resembling ABL, this patient was not classified as ABL but as AML with basophilia according to consensus criteria [8].

So far, it remains unknown whether patients with CBL have a better prognosis and may respond better to antineoplastic drugs compared to those with ABL. In 1 of our patients with CBL, disease stabilization was reached during HU therapy for more than 30 mo. However, our second patients with CBL died after 3 mo. We believe that more data and more patients, preferably collected in a multi-national registry study, are required to define the precise prognosis of patients with secondary CBL and secondary ABL.

Basophils and mast cells share several different antigens and cellular functions [31−33,[38], [39], [40], [41]]. Therefore, it is of great importance to confirm the nature of a basophilic leukemia by immunological phenotyping [38−41]. Basophils usually display several myeloid antigens, including CD11b as well as CD123 and the basophil-related antigens CD203c, BB1 and 2D7, but do not express substantial amounts of KIT or chymase [[38], [39], [40], [41], [42], [43], [44]]. By contrast, mast cells display substantial amounts of tryptase and KIT and some CD203c, but usually do not exhibit larger amounts of CD123 unless mast cells are very immature. In our patients, neoplastic cells were found to display CD11b, CD18, and CD123 as well as BB1, 2D7 and low amounts of KIT, but did not express chymase, confirming that these cells are basophils but not mast cells. Expression of KIT confirms that these cells were rather immature basophils and/or basophilic precursor cells.

We also examined the expression of additional cell surface antigens that might serve as therapeutic targets or as indicators of drug resistance on leukemic CD203c+ basophils. In these studies, we found that leukemic basophils express the cell surface targets CD33 (Siglec-3), CD123 and CD371 (CLL-1) as well as the immune checkpoint antigen CD274 (PD-L1). No major differences were found when comparing the phenotypes of basophils obtained from different donors, CBL vs ABL, or various organ sources, including the BM, peripheral blood and spleen.

Basophils and mast cells also share functional IgE-receptors [38,40]. However, whereas fully functional IgE receptors are detected quite early in basophil development, immature mast cell progenitors usually lack functional IgE-binding sites [45,46]. In the present study, we were able to detect IgE-binding sites on leukemic basophils and confirmed that these cells express fully functional receptors, as cross-linking of these receptors resulted in a dose-dependent release of histamine from basophils. The variability in releasability of basophils found among the samples tested is a well-known phenomenon. In fact, as in healthy individuals, we were able to classify our patients into good releasers (n = 2) and poor releasers (n = 2). An alternative explanation may be that basophils were functionally immature cells lacking a fully functional IgE receptor or all downstream signaling molecules and cascades required to actively release histamine. Finally, it is well known that certain genetic background-variables, such as Syk expression and Syk deficiency, are associated with a particular potential of basophils to release histamine [47,48].

An interesting aspect is that basophils produce a number of pathogenically relevant mediators and cytokines which may contribute to the evolution and/or progression of a myeloid neoplasm [9,18]. For example, basophils reportedly express and release vascular endothelial growth factor and hepatocyte growth factor, both of which may contribute to the increased angiogenesis and tissue remodeling in myeloid neoplasms [9,18]. Whereas the prognostic impact of basophilia is well established in these malignancies, the active pathogenic role of basophils in these neoplasms may be underestimated [9]. Whether basophil-derived mediators and cytokines also play a role in disease progression and resistance in patients with CBL and ABL remains to be elucidated.

A number of previous and more recent data suggest that basophil lineage expansion in various myeloid neoplasms is associated with a poor prognosis concerning survival and progression-free survival [49,50]. In addition, it is well known that patients with secondary ABL or CBL have a poor prognosis. In the current study, we made a corresponding observation. In fact, all 5 patients with basophilic leukemia (basophilic crisis) progressed after a variable latency period and all died from their disease within 2.5 Y. In addition, resistance against conventional antineoplastic drugs, including HU and demethylating agents was seen in all patients.

So far, no effective standard treatments for patients with CBL or ABL are available. An exception may be BCR-ABL1+ secondary CBL where BCR-ABL1-targeting drugs may be sufficient to bring the disease process under control. In other patients, including those who suffer from MDS, MPN or MPN/MDS with secondary CBL or ABL, hypomethylating agents or polychemotherapy may be reasonable therapeutic options. However, so far, no controlled clinical studies evaluating the efficacy of these drugs in patients with CBL and ABL have been conducted. Our data suggest that neoplastic cells in patients with secondary CBL or ABL in the MDS/MPN/AML context are often refractory to all these therapies. Even the repeated administration of hypomethylating agents did not lead to clinically meaningful responses in these patients.

One novel approach to overcome resistance of MDS/MPN/AML cells against demethylating agents is to combine these drugs with venetoclax, a BCL-2 inhibitor [51]. This drug combination has recently been described to be efficacious in patients with advanced MDS and AML who are not eligible for poly-chemotherapy. In our patients, the demethylating drug azacitidine was administered as single drug but not in combination with venetoclax. However, in our *in vitro* experiments, this drug combination was found to work in basophil-committed progenitor cells and in primary patient-derived cells. Based on this observation it is tempting to speculate that this drug combination may also work in patients with CBL or ABL. However, whether these drugs can indeed induce long lasting responses or even remissions in these patients remains to be determined in clinical studies.

We also tested the effects of other drugs on *in vitro* proliferation of neoplastic basophils, the BCR-ABL1+ basophil cell line KU812 and in primary cells derived from patients with CBL or ABL. As expected the BCR-ABL1 inhibitors produced superior growth-inhibitory effects in KU812 cells. In addition, these drugs were also effective in some of the patient samples tested. Overall, however, the drugs applied showed variable effects on patient-derived cells, and the response patterns differed from patient to patient. This is best explained by the heterogeneous target expression profiles in these cells and would argue for personalized *in vitro* drug testing in individual patients and the application of drug combinations. Indeed, personalized drug testing prior to chemotherapy or targeted drug therapy has recently shown to be of value and to predict responses of neoplastic cells in these patients. However, it remains unknown whether such approach would also support the development of better more effective therapies in patients with CBL and ABL. One additional target that we identified but did not test in this study was CD33. However, although it is well known that basophils display CD33 and may respond to CD33-targeted drugs [38,52], the therapeutic window is small because normal stem and progenitor cells also express CD33.

Together, we present 5 patients with Ph-negative basophilic leukemia or a related myeloid disorder. In all 5 patients, basophil involvement was confirmed by morphologic and phenotypic studies, and functional analyses. Treatment with various cytostatic drugs, including HU and azacitidine, did not result in sustained treatment responses. However, *in vitro* studies performed with various targeted drugs and drug combinations using patient-derived cells and the basophilic leukemia cell line KU812 suggest that the combination azacitidine+venetoclax may be an effective approach to suppress growth and survival of neoplastic basophils in these disorders. Whether this combination is also effective *in vivo* in patients remains to be determined in clinical trials.

## Acknowledgments

We like to thank Anna-Katharina Schruef, Nadine Witzeneder, and Christian Milosits for their skillful technical assistance.

## Author contributions

DB: Conceptualization, Formal analysis, Investigation, Writing - Original Draft, Visualization. KB: Investigation. CK: Conceptualization, Resources. SG: Investigation. GS: Investigation. DS: Investigation. CS: Resources. PB: Resources, PK: Resources, AIS: Resources, GG: Investigation. RT: Investigation. GH: Resources. IS: Investigation, Validation. PBS: Methodology, Resources. WRS: Formal analysis, Resources. PV: Conceptualization, Methodology, Resources, Writing - Review & Editing, Supervision.

## Appendix Supplementary materials

Image, application 1

## References

[1] Denburg JA, Browman G. (1988). Prognostic implications of basophil differentiation in chronic myeloid leukemia. Am J Hematol.

[2] Yamauchi K, Arimori S. (1990). Basophilic crisis in chronic myelogenous leukemia: case report and literature review in Japan. Jpn J Med.

[3] Hasford J, Pfirrmann M, Hehlmann R, Allan NC, Baccarani M, Kluin-Nelemans JC (1998). A new prognostic score for survival of patients with chronic myeloid leukemia treated with interferon alfa. Writing Committee for the Collaborative CML Prognostic Project Group. J Natl Cancer Inst.

[4] Steegmann JL, Odriozola J, Rodriguez-Salvanés F, Giraldo P, García-Laraña J, Ferro MT (1999). Stage, percentage of basophils at diagnosis, hematologic response within six months, cytogenetic response in the first year: the main prognostic variables affecting outcome in patients with chronic myeloid leukemia in chronic phase treated with interferon-alpha. Results of the CML89 trial of the Spanish Collaborative Group on interferon-alpha2a and CML. Haematologica.

[5] Sugimoto N, Ishikawa T, Gotoh S, Shinzato I, Matsushita A, Nagai K (2004). Primary myelofibrosis terminated in basophilic leukemia and successful allogeneic bone marrow transplantation. Int J Hematol.

[6] Wimazal F, Baumgartner C, Sonneck K, Zauner C, Geissler P, Schur S (2008). Mixed-lineage eosinophil/basophil crisis in MDS: a rare form of progression. Eur J Clin Invest.

[7] Pidala J, Pinilla-Ibarz J, Cualing HD. (2008). A case of acute basophilic leukemia arising from chronic myelogenous leukemia with development of t(7;8)(q32;q13). Cancer Genet Cytogenet.

[8] Valent P, Sotlar K, Blatt K, Hartmann K, Reiter A, Sadovnik I, Sperr WR, Bettelheim P, Akin C, Bauer K (2017). Proposed diagnostic criteria and classification of basophilic leukemias and related disorders. Leukemia.

[9] Valent P, Horny HP, Arock M. (2018). The underestimated role of basophils in Ph(+) chronic myeloid leukaemia. Eur J Clin Invest.

[10] Lübbert M, Suciu S, Baila L, Rüter BH, Platzbecker U, Giagounidis A, Selleslag D, Labar B, Germing U, Salih HR (2011). Low-dose decitabine versus best supportive care in elderly patients with intermediate- or high-risk myelodysplastic syndrome (MDS) ineligible for intensive chemotherapy: final results of the randomized phase III study of the European Organisation for Research and Treatment of Cancer Leukemia Group and the German MDS Study Group. J Clin Oncol.

[11] Kuendgen A, Müller-Thomas C, Lauseker M, Haferlach T, Urbaniak P, Schroeder T, Brings C, Wulfert M, Meggendorfer M, Hildebrandt B (2018). Efficacy of azacitidine is independent of molecular and clinical characteristics - an analysis of 128 patients with myelodysplastic syndromes or acute myeloid leukemia and a review of the literature. Oncotarget.

[12] Germing U, Schroeder T, Kaivers J, Kündgen A, Kobbe G, Gattermann N. (2019). Novel therapies in low- and high-risk myelodysplastic syndrome. Expert Rev Hematol.

[13] Pollyea DA, Amaya M, Strati P, Konopleva MY. (2019). Venetoclax for AML: changing the treatment paradigm. Blood Adv.

[14] Arber DA, Orazi A, Hasserjian R, Thiele J, Borowitz MJ, Le Beau MM, Bloomfield CD, Cazzola M, Vardiman JW (2016). The 2016 revision to the World Health Organization classification of myeloid neoplasms and acute leukemia. Blood.

[15] Stevens-Kroef M, Simons A, Rack K, Hastings RJ. (2017). Cytogenetic nomenclature and reporting. Methods Mol Biol.

[16] Valent P, Besemer J, Muhm M, Majdic O, Lechner K, Bettelheim P. (1989). Interleukin 3 activates human blood basophils via high-affinity binding sites. Proc Natl Acad Sci (USA).

[17] Kishi K. (1985). A new leukemia cell line with Philadelphia chromosome characterized as basophil precursors. Leuk Res.

[18] Cerny-Reiterer S, Ghanim V, Hoermann G, Aichberger KJ, Herrmann H, Muellauer L, Repa A, Sillaber C, Walls AF, Mayerhofer M (2012). Identification of basophils as a major source of hepatocyte growth factor in chronic myeloid leukemia: a novel mechanism of BCR-ABL1-independent disease progression. Neoplasia.

[19] Mueller N, Wicklein D, Eisenwort G, Jawhar M, Berger D, Stefanzl G, Greiner G, Boehm A, Kornauth C, Muellauer L (2018). CD44 is a RAS/STAT5-regulated invasion receptor that triggers disease expansion in advanced mastocytosis. Blood.

[20] Hauswirth AW, Natter S, Ghannadan M, Majlesi Y, Schernthaner GH, Sperr WR, Bühring HJ, Valenta R, Valent P. (2002). Recombinant allergens promote expression of CD203c on basophils in sensitized individuals. J Allergy Clin Immunol.

[21] Smiljkovic D, Kiss R, Lupinek C, Hoermann G, Greiner G, Witzeneder N, Krajnik G, Trautinger F, Vrtala S, Mittermann I (2020). Microarray-based detection of allergen-reactive IgE in patients with mastocytosis. J Allergy Clin Immunol Pract.

[22] Gupta R, Jain P, Anand M. (2004). Acute basophilic leukemia: case report. Am J Hematol.

[23] Tang G, Woods LJ, Wang SA, Brettler D, Andersen M, Miron PM (2009). Chronic basophilic leukemia: a rare form of chronic myeloproliferative neoplasm. Hum Pathol.

[24] Shvidel L, Shaft D, Stark B, Shtalrid M, Berrebi A, Resnitzky P. (2003). Acute basophilic leukaemia: eight unsuspected new cases diagnosed by electron microscopy. Br J Haematol.

[25] Xue YQ, Guo Y, Lu DR, Gu J, Lu DW, Gong JX (1991). A case of basophilic leukemia bearing simultaneous translocations t(8;21) and t(9;22). Cancer Genet Cytogenet.

[26] Rojas-Atencio A, Urdaneta K, Soto-Quintana M, Alvarez Nava F, Cañizales J, Solis E (2011). Trisomy 19 and t(9;22) in a patient with acute basophilic leukemia. Case Rep Pathol.

[27] Servitzoglou M, Grenzelia M, Baka M, Harisi M, Pourtsidis A, Bouhoutsou D (2014). A novel karyotype in acute myeloid leukemia with basophilia. Pediatr Hematol Oncol.

[28] Kritharis A, Brody J, Koduru P, Teichberg S, Allen SL. (2011). Acute basophilic leukemia associated with loss of gene ETV6 and protean complications. J Clin Oncol.

[29] Quelen C, Lippert E, Struski S, Demur C, Soler G, Prade N (2011). Identification of a transforming MYB-GATA1 fusion gene in acute basophilic leukemia: a new entity in male infants. Blood.

[30] Peterson LC, Parkin JL, Arthur DC, Brunning RD. (1991). Acute basophilic leukemia. A clinical, morphologic, and cytogenetic study of eight cases. Am J Clin Pathol.

[31] Shin SY, Koo SH, Kwon KC, Park JW, Ko CS, Jo DY. (2007). Monosomy 7 as the sole abnormality of an acute basophilic leukemia. Cancer Genet Cytogenet.

[32] Duchayne E, Demur C, Rubie H, Robert A, Dastugue N (1999). Diagnosis of acute basophilic leukemia. Leuk Lymphoma.

[33] Nissenblatt MJ. (1980). Basophilic transformation of chronic myelogenous leukemia. South Med J.

[34] Denburg JA, Wilson WE, Bienenstock J. (1982). Basophil production in myeloproliferative disorders: increases during acute blastic transformation of chronic myeloid leukemia. Blood.

[35] Muehleck SD, McKenna RW, Arthur DC, Parkin JL, Brunning RD. (1984). Transformation of chronic myelogenous leukemia: clinical, morphologic, and cytogenetic features. Am J Clin Pathol.

[36] Yamauchi K, Arimori S. (1990). Basophilic crisis in chronic myelogenous leukemia: case report and literature review in Japan. Jpn J Med.

[37] Arber D, Brunning RD, Orazi A, Porwit A, Peterson LC, Thiele J, Le Beau MM, Hasserjian RP. Acute myeloid leukemia – not otherwise specified. In: Swerdlow SH, Campo E, Harris NL, Jaffe ES, Pileri SA, Stein H, Thiele J, Arber DA, Hasserjian RP, Le Beau MM, Orazi A, Siebert R. WHO Classification of Tumours of Haematopoietic and Lymphoid Tissues. IARC Press, Lyon, France. pp 156-165.

[38] Valent P, Bettelheim P. (1992). Cell surface structures on human basophils and mast cells: biochemical and functional characterization. Adv Immunol.

[39] Agis H, Beil WJ, Bankl HC, Füreder W, Sperr WR, Ghannadan M (1996). Mast cell-lineage versus basophil lineage involvement in myeloproliferative and myelodysplastic syndromes: diagnostic role of cell-immunophenotyping. Leuk Lymphoma.

[40] Valent P, Schernthaner GH, Sperr WR, Fritsch G, Agis H, Willheim M (2001). Variable expression of activation-linked surface antigens on human mast cells in health and disease. Immunol Rev.

[41] Escribano L, Díaz-Agustín B, Bellas C, Navalón R, Nuñez R, Sperr WR (2001). Utility of flow cytometric analysis of mast cells in the diagnosis and classification of adult mastocytosis. Leuk Res.

[42] Agis H, Krauth MT, Mosberger I, Müllauer L, Simonitsch-Klupp I, Schwartz LB, Böhm A, Fritsch G, Horny HP, Valent P (2006). Enumeration and immunohistochemical characterisation of bone marrow basophils in myeloproliferative disorders using the basophil specific monoclonal antibody 2D7. J Clin Pathol.

[43] Agis H, Krauth MT, Böhm A, Mosberger I, Müllauer L, Simonitsch-Klupp I, Walls F, Horny HP, Valent P (2006). Identification of basogranulin (BB1) as a novel immunohistochemical marker of basophils in normal bone marrow and patients with myeloproliferative disorders. Am J Clin Pathol.

[44] Füreder W, Schernthaner GH, Ghannadan M, Hauswirth A, Sperr WR, Semper H, Majlesi Y, Zwirner J, Götze O, Bühring HJ (2001). Quantitative, phenotypic, and functional evaluation of basophils in myelodysplastic syndromes. Eur J Clin Invest.

[45] Valent P, Schmidt G, Besemer J, Mayer P, Zenke G, Liehl E, Hinterberger W, Lechner K, Maurer D, Bettelheim P. (1989). Interleukin-3 is a differentiation factor for human basophils. Blood.

[46] Schernthaner GH, Hauswirth AW, Baghestanian M, Agis H, Ghannadan M, Worda C, Krauth MT, Printz D, Fritsch G, Sperr WR (2005). Detection of differentiation- and activation-linked cell surface antigens on cultured mast cell progenitors. Allergy.

[47] Kepley CL, Youssef L, Andrews RP, Wilson BS, Oliver JM. (1999). Syk deficiency in nonreleaser basophils. J Allergy Clin Immunol.

[48] Kepley CL, Youssef L, Andrews RP, Wilson BS, Oliver JM. (2000). Multiple defects in Fc epsilon RI signaling in Syk-deficient nonreleaser basophils and IL-3-induced recovery of Syk expression and secretion. J Immunol.

[49] Matsushima T, Handa H, Yokohama A, Nagasaki J, Koiso H, Kin Y, Tanaka Y, Sakura T, Tsukamoto N, Karasawa M (2003). Prevalence and clinical characteristics of myelodysplastic syndrome with bone marrow eosinophilia or basophilia. Blood.

[50] Wimazal F, Germing U, Kundi M, Noesslinger T, Blum S, Geissler P, Baumgartner C, Pfeilstoecker M, Valent P, Sperr WR. (2010). Evaluation of the prognostic significance of eosinophilia and basophilia in a larger cohort of patients with myelodysplastic syndromes. Cancer.

[51] Liu B, Guo Y, Deng L, Qiao Y, Jian J. (2020). The efficacy and adverse events of venetoclax in combination with hypomethylating agents treatment for patients with acute myeloid leukemia and myelodysplastic syndrome: a systematic review and meta-analysis. Hematology.

[52] Krauth MT, Böhm A, Agis H, Sonneck K, Samorapoompichit P, Florian S, Sotlar K, Valent P. (2007). Effects of the CD33-targeted drug gemtuzumab ozogamicin (mylotarg) on growth and mediator secretion in human mast cells and blood basophils. Exp Hematol.

