# Supplementary material for: Secondary basophilic leukemia in Ph-negative myeloid neoplasms: A distinct subset with poor prognosis
Source: Neoplasia. 2021 Oct 31;23(12):1183–91. doi: 10.1016/j.neo.2021.09.010 (PMC8572856; doi:10.1016/j.neo.2021.09.010)
Supplement: Supplementary file 1 [file mmc1.docx]

Supplement to:

**Secondary basophilic leukemia in Ph-negative myeloid neoplasms: a distinct subset with poor prognosis**

Daniela Berger,^1^ Karin A. Bauer^1,2^, Christoph Kornauth^1,2,3^, Susanne Gamperl^1^, Gabriele Stefanzl^1^, Dubravka Smiljkovic^1^, Christian Sillaber^1^, Peter Bettelheim^4^, Paul Knöbl^1^, Ana-Iris Schiefer^3^, Georg Greiner^2,5,6^, Renate Thalhammer^6^, Gregor Hoermann^2,6,7^, Ilse Schwarzinger^6^, Philipp B. Staber^1^,

Wolfgang R. Sperr^1^, Peter Valent^1^

^1^Department of Internal Medicine I, Division of Hematology and Hemostaseology, Medical University of Vienna, Austria; ^2^Ludwig Boltzmann Institute for Hematology and Oncology (LBI HO), Medical University of Vienna, Austria; ^3^Department of Pathology, Medical University of Vienna, Austria; ^4^Division of Hematology and Oncology, Elisabethinen Hospital Linz and Europa-Platz Labor Linz, Austria;

^5^Ihr Labor, Medical Diagnostic Laboratories, Vienna, Austria; ^6^Department of Laboratory Medicine, Medical University of Vienna, Austria;

^7^Munich Leukemia Laboratory (MLL), Munich, Germany.

**Supplemental Table S1**

**Specification of antibodies used in immunohistochemistry and immunocytochemistry studies**

| **Antigen** | **Clone** | **Ig class** | **Animal source** | **Provider/Company*** |
| --- | --- | --- | --- | --- |
| Basogranulin | BB1** | IgG | Mouse | University of Southampton |
| Ki-1 / CD30 | Ber-H2 | IgG | Mouse | Dako |
| KIT / CD117 | polyclonal | - | Rabbit | Dako |
| Chymase | B7 | IgG | Mouse | Millipore |
| 2D7 | 2D7 | IgG1 | Mouse | Biolegend |
| Tryptase | G3 | IgG | Mouse | Cell Marque |

Abbreviations: CD, cluster of differentiation; IgG, immunoglobulin G.

*Company location: Biolegend, San Diego, CA, USA; Cell Marque, Rocklin, CA, USA; Dako, Glostrup, Denmark; Millipore, Burlington, MA, USA.

**The BB1 antibody was kindly provided by Andrew Walls (University of Southampton).

**Supplemental Table S2**

**Specification of antibodies used in flow cytometry staining experiments**

| **Antigen** | **CD** | **Clone** | **Isotype** | **Conjugate** | **Source*** |
| --- | --- | --- | --- | --- | --- |
| LFA-2 | CD2 | RPA-2.10 | mIgG1 | PE | BD |
| Tetraspanin-29 | CD9 | M-L13 | mIgG1 | PE | BD |
| C3biR | CD11b | ICRF44 | mIgG1 | PE | Biolegend |
| Aminopeptidase-N | CD13 | WM15 | mIgG1 | PE | BD |
| LPS receptor | CD14 | TÜK4 | mIgG1 | FITC | Dako |
| Integrin beta-2 | CD18 | 6.7 | mIgG1 | PE | BD |
| IL-2RA | CD25 | 2A3 | mIgG1 | PE | BD |
| DPPIV | CD26 | M-A261 | mIgG1 | PE | BD |
| Ki-1 | CD30 | BerH8 | mIgG1 | PE | BD |
| Siglec-3 | CD33 | WM53 | mIgG1 | PE | BD |
| LCA | CD45 | 2D1 | mIgG1 | PerCP | BD |
| Tetraspanin-30 | CD63 | CLBGran/12 | mIgG1 | PE | Beckman Coulter |
| GM-CSFR alpha | CD116 | hGMCSFR-M1 | mIgG1 | PE | BD |
| SCFR/KIT | CD117 | 104D2 | mIgG1 | PE | BD |
| IL-3RA | CD123 | AC145 | mIgG1 | APC | Miltenyi Biotec |
| IL-3RA | CD123 | 7G3 | mIgG2A | PE | BD |
| Sialomucin | CD164 | N6B6 | mIgG2A | PE | BD |
| ENPP3 | CD203c | FR3-16A11 | mIgG1 | APC | Miltenyi Biotec |
| ENPP3 | CD203c | 97A6 | mIgG2A | PE | Beckman Coulter |
| PD-L1 | CD274 | 29E.2A3 | mIgG2B | PE | Biolegend |
| PD1 | CD279 | EH12.2H7 | mIgG1 | PE | Biolegend |
| Siglec-6 | CD327 | 767329 | mIgG2A | PE | R&D Systems |
| CLL-1 | CD371 | 50C1 | mIgG2A | PE | BD |
| FcεR1A | n.c. | AER-32 | mIgG2B | APC | Biolegend |
| IL-1RAP | n.c. | 89412 | mIgG1 | - | R&D Systems |

Abbreviations: CD, cluster of differentiation; LFA-2, lymphocyte function-associated antigen 1; mIgG, mouse immunoglobulin G; PE, phycoerythrin; BD, Becton Dickinson; FITC, fluorescein isothiocyanate; IL-2RA, [interleukin 2](https://en.wikipedia.org/wiki/Interleukin_2) receptor alpha; DPPIV, dipeptidylpeptidase 4; TNFRSF8, tumor necrosis factor receptor superfamily member 8; Siglec-3, sialic acid binding immunoglobulin like lectin 3; LCA, leukocyte common antigen; PerCP, peridinin chlorophyll protein; GM-CSFR alpha, granulocyte-macrophage colony-stimulating factor receptor alpha; SCFR, stem cell factor receptor; IL-3RA, interleukin-3 receptor alpha; APC, allophycocyanin; ENPP3, ectonucleotide pyrophosphatase/phosphodiesterase 3; PD-L1, programmed cell death 1 ligand 1; PD-1, programmed cell death protein 1; Siglec-6, sialic acid binding immunoglobulin like lectin 6; CLL-1, C-type lectin-like molecule-1; IL-1RAP, interleukin-1 receptor accessory protein; n.c., not clustered.

*Company location: BD Biosciences, San Jose, CA, USA; Biolegend, San Diego, CA, USA; Dako, Glostrup, Denmark; Beckman Coulter, Krefeld, Germany; Miltenyi Biotec, Bergisch Gladbach, Germany; R&D Systems, Minneapolis, MN, USA.

**Supplemental Table S3**

**Karyotype abnormalities detected in neoplastic cells**

-----------------------------------------------------------------------------------------------------------------

Patient # Karyotype*

-----------------------------------------------------------------------------------------------------------------

#1 46,XY

#2 47,XY,der(7)t(X;7)(p?;q1?)del(q1?),+8,?del(11)(?),del(20)(q1?1)

#3 47,XY,del(5)(q14),t(5;12)(q31;p13),add(8)(q?11),+add(11)(p14)

?del(13)(q13q21),add(17)(q11)

#4 46,XX,del(11)(q23.3),der(14)t(4;14)(q?;q?)

#5 46,XX

--------------------------------------------------------------------------------------------------------

*The karyotype was determined in bone marrow leukocytes by conventional karyotyping and/or (multi-color) fluorescence in situ hybridization (FISH).

**Supplemental Table S4**

**Mutation analyses in patients**

| **Mutated Gene** |  | **Mutations detected in Patient #** | | |  |
| --- | --- | --- | --- | --- | --- |
|  | **#1** | **#2** | **#3** | **#4** | **#5** |
| *BCR-ABL1* | - | - | - | - | - |
| *FIP1L1-PDGFRA* | - | n.d. | n.d. | n.d. | - |
| *KIT* D816V | - | - | - | - | - |
| *JAK2* V617F | - | - | - | + | - |
| *CSF3R* | - | + | - | - | - |
| *MPL* | + | - | - | - | - |
| *FLT3* ITD | - | - | - | - | - |
| *RUNX1* | - | + | - | - | - |
| *CEBPA* | - | - | - | - | + |
| *KRAS* | - | - | - | - | + |
| *CBL* | - | + | - | + | - |
| *NPM1* | - | - | - | - | - |
| *PTPN11* | - | - | - | + | - |
| *IDH1* | - | + | - | - | - |
| *ASXL1* | - | - | - | + | - |
| *SRSF2* | + | - | - | - | + |
| *TP53* | - | - | +* | - | - |
| *TET2* | - | - | - | - | + |

Gene mutations were identified in neoplastic cells (bone marrow or blood) of patients with basophilia by next generation sequencing (NGS) and/or qPCR. Variant allele frequencies (VAF) >2% were considered as a positive result (+) and VAF <2% as a negative result (-). Abbreviations: *PDGFRA*, platelet-derived growth factor receptor alpha; *JAK2*, Janus kinase 2; *CSF3R*, colony-stimulating factor 3 receptor; *FLT3*, fms related receptor tyrosine kinase 3; *KRAS*, Kirsten rat sarcoma viral oncogene homolog; *CBL*, Casitas B-lineage lymphoma; *NPM1*, nucleophosmin 1; *PTPN11*, protein tyrosine phosphatase non-receptor type 11; *IDH1*, isocitrate dehydrogenase; *ASXL1*, ASXL transcriptional regulator 1; *SRSF2*, serine and arginine rich splicing factor 2; *TP53*, tumor protein p53; n.d., not determined. *In this patient, two *TP53* mutations (exon 7: C722T and exon 5: G481A) were detected in neoplastic cells.

**Supplemental Table S5**

**Effects of antineoplastic drugs on proliferation of KU812 cells**

| **Drug** | **Mode of action** | **Concentration range (µM)** | **KU812 IC_50_ range (µM)** |
| --- | --- | --- | --- |
| Azacitidine | Demethylating agent | 0.5-100 | 1-5 |
| Cladribine | Inhibition of DNA synthesis | 0.001-10 | 0.5-1 |
| Cytarabine | Inhibition of DNA synthesis | 0.001-10 | 0.01-0.05 |
| Dasatinib** | TKI | 0.00001-0.001 | 0.0001-0.0005 |
| Hydroxyurea | RNR inhibitor | 5-5000 | 100-500 |
| JQ1 | BET inhibitor | 0.001-10 | 0.1-0.5 |
| Ponatinib** | TKI | 0.00001-0.001 | 0.0001-0.0005 |
| Rapamycin | mTOR | 0.001-10 | >10 |
| Selinexor | SINE | 0.001-10 | 0.1-0.5 |
| Venetoclax | BCL-2 inhibitor | 0.01-10 | 5-10 |

Abbreviations: BET, bromodomain and extra-terminal; DNMT, DNA methyltransferase; mTOR, mammalian target of rapamycin; SINE, Selective Inhibitor of Nuclear Export; RNR, ribonucleotide reductase; TKI, tyrosine kinase inhibitor; IC_50_, half maximal inhibitory concentration, assessed by ^3^H-thymidine uptake experiments in KU812 cells; ** KU812 is a BCR-ABL positive cell line and therefore responds to the TKI dasatinib and ponatinib.

**Supplemental Table S6**

| **Drug applied** | **IC_50_** **(µM) obtained with neoplastic cells in:** | | |
| --- | --- | --- | --- |
|  | **Patient #1** | **Patient #2** | **Patient #4** |
| Azacitidine | n.d. | 1-5 | 1-5 |
| Cladribine | n.d. | n.d. | 0.1-0.5 |
| Cytarabine | n.d. | 0.01-0.05 | 0.01-0.05 |
| Dasatinib | 0.01-0.05 | n.d. | >10 |
| Hydroxyurea | n.d. | 100-500 | 50-100 |
| JQ1 | 0.1-0.5 | n.d. | 1-5 |
| Ponatinib | 0.1-0.5 | n.d. | 1-5 |
| Rapamycin | n.d. | n.d. | 0.1-0.5 |
| Ruxolitinib | n.d. | n.d. | 0.1-0.5 |
| Selinexor | n.d. | n.d. | 0.05-0.1 |
| Venetoclax | n.d. | 1-5 | 1-5 |

**Growth-inhibitory effects of various drugs on proliferation of neoplastic cells derived from patients with basophilic leukemia**

IC_50_, half maximal inhibitory concentration, assessed by ^3^H-thymidine uptake experiments using cells obtained from patient #1, #2 and #4. Abbreviations: n.d., not determined.

**Supplemental Figures**


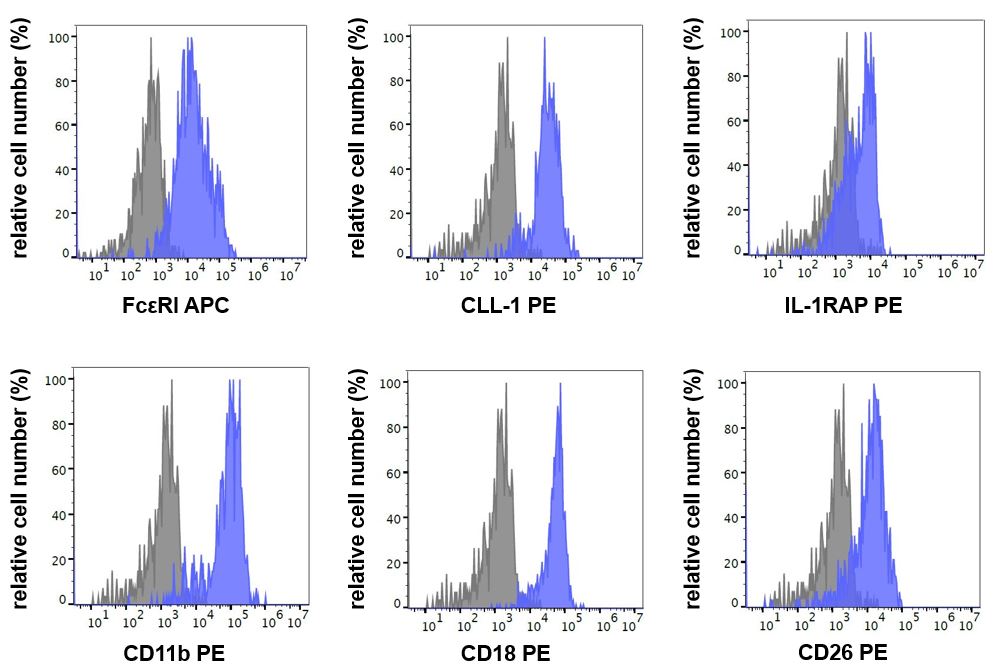


**Supplemental Figure S1**

**Expression of surface molecules on CD203c^+^/CD14^–^ basophils in patients #3**

CD203c^+^/CD14^–^ basophils obtained from a patient with acute basophilic leukemia (#3) were stained with monoclonal antibodies against FcεRI, CLL-1, IL1RAP, CD11b, CD18 and CD26 (blue histograms). Isotype-matched antibodies were used as a control (grey histograms).

**
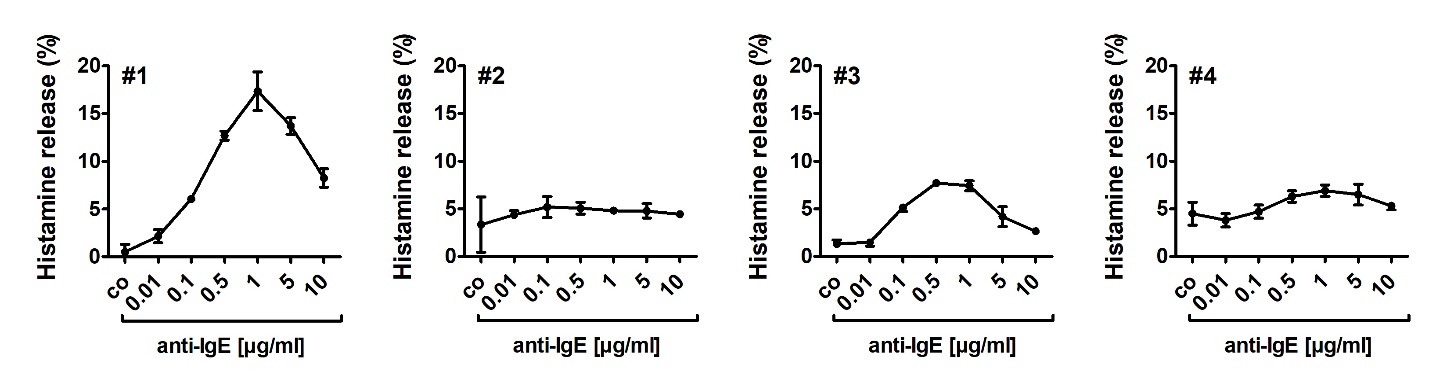
**

**Supplemental Figure S2A**

**IgE-dependent histamine release in primary patient-derived cells**

Dextran-enriched basophils from four patients with basophilia (#1, #2, #3, #4) were incubated in histamine release buffer in the absence (co) or presence of the polyclonal anti-IgE antibody (0.01-10 µg/ml) at 37°C for 30 minutes. After incubation, cells were centrifuged, and the cell-free supernatants and the cell suspensions were analyzed for histamine content. Histamine release is expressed as percentage of total (cellular+extracellular) histamine. Results represent the mean±SD of triplicates. Numbers of patients (#) refer to the identification numbers shown in Table 1.

**
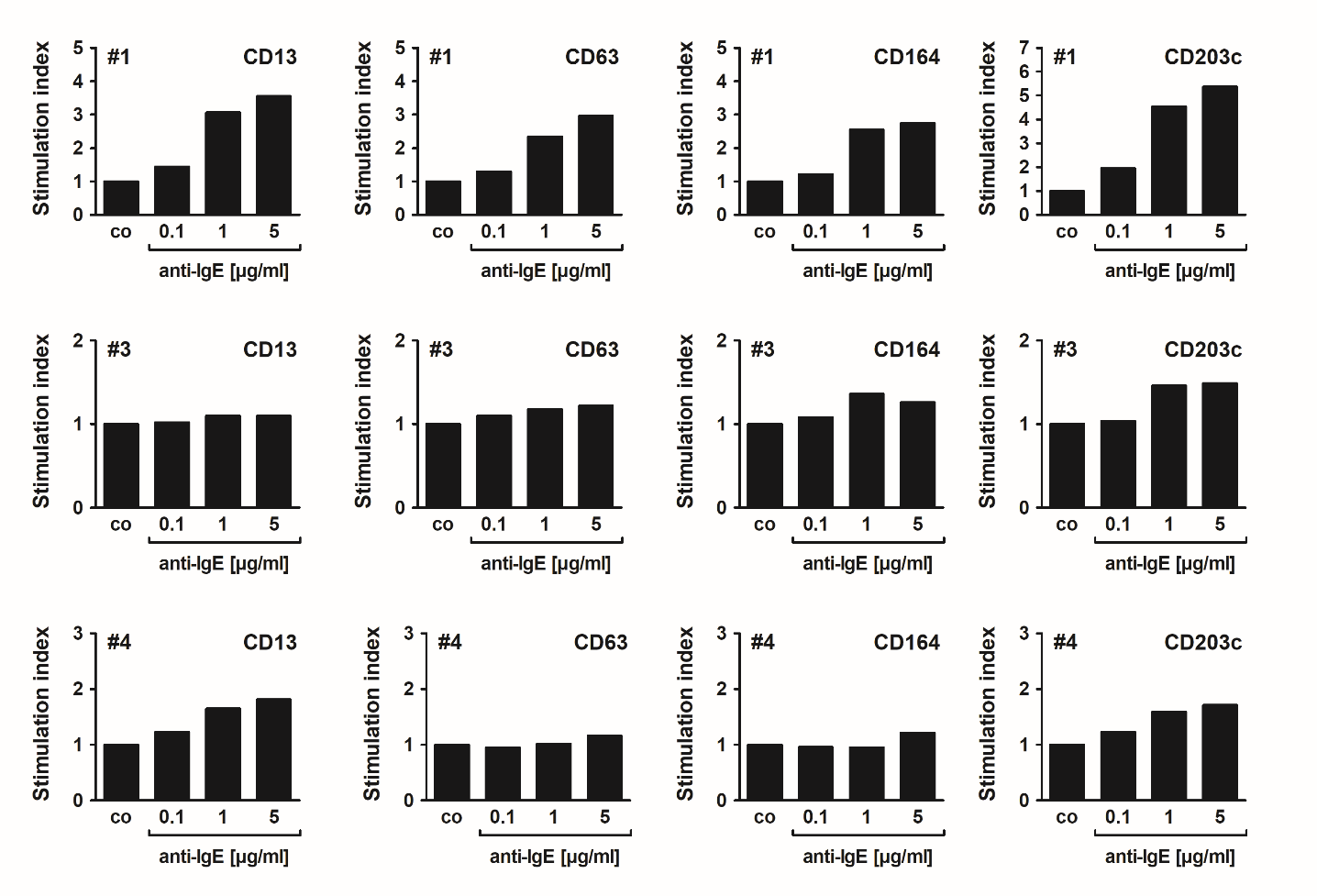
**

**Supplemental Figure S2B**

**IgE-mediated upregulation of activation-linked cell surface antigens on basophils**.

Peripheral blood leukocytes obtained from patients #1, #3 and #4 were exposed to the polyclonal anti-IgE antibody (0.1-5 µg/ml) at 37°C for 15 minutes. Then, cells were stained with monoclonal antibodies against CD13, CD63, CD164 or CD203c (15 minutes, 37°C) and analyzed by multicolor flow cytometry. Basophils were defined as CD203c+ cells. Anti-IgE-induced upregulation of CD antigens was determined from median fluorescence intensities (MFI) obtained with stimulated (MFI_stim_) and unstimulated (MFI_control_) cells and expressed as stimulation index (SI=MFI_stim_: MFI_control_). Numbers of patients (#) refer to the identification numbers shown in Table 1.


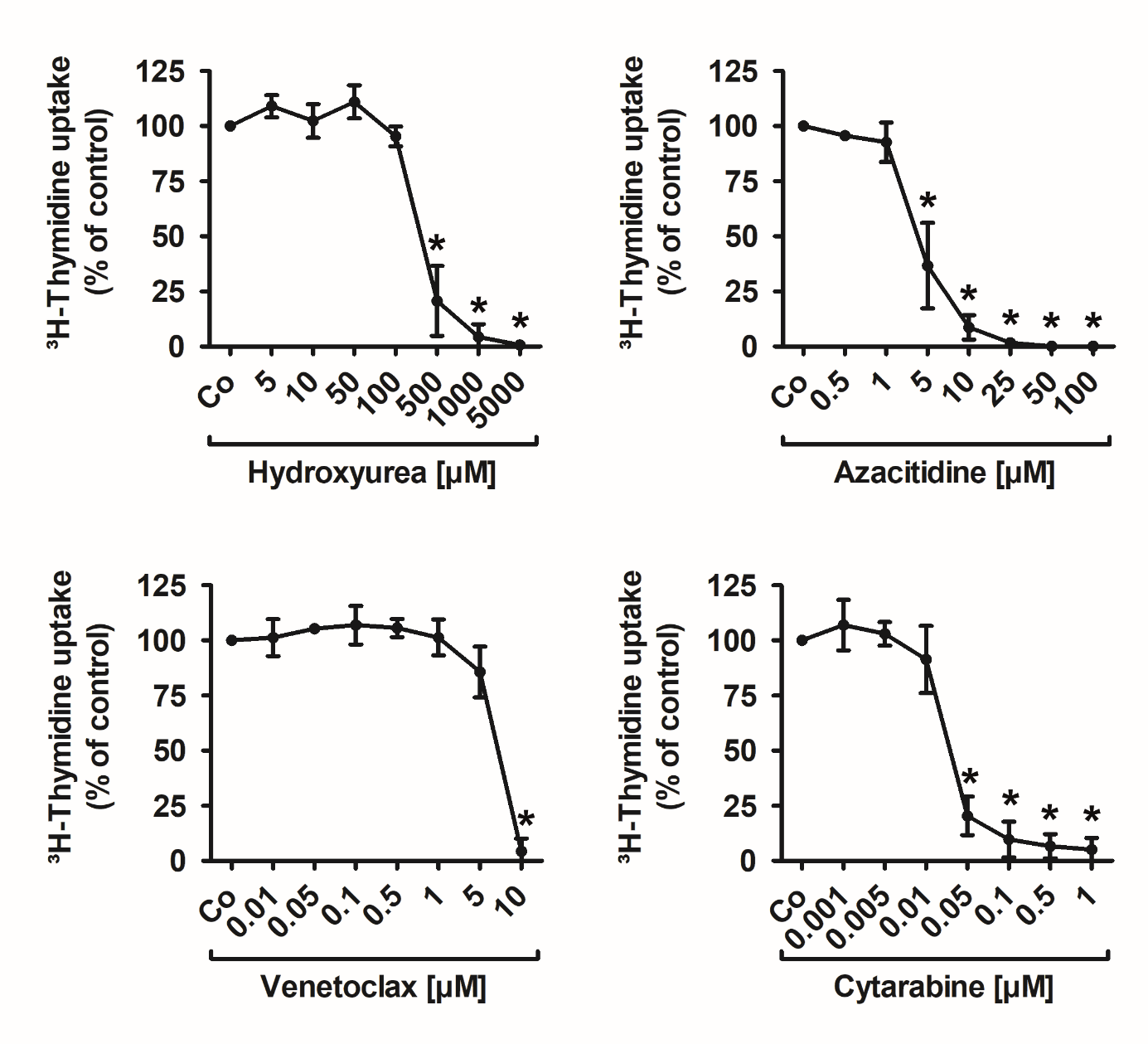


**Supplemental Figure S3**

**Effects of targeted drugs on proliferation of KU812 cells**

KU812 cells were incubated in control medium (Co) or in medium containing various concentrations of azacitidine, cytarabine, hydroxyurea, or venetoclax (as indicated) at 37°C for 48 hours. Proliferation was determined by measuring ^3^H-thymidine uptake. Results are expressed as percent of control and represent the mean±SD from three independent experiments. * P<0.05 compared to control.

**
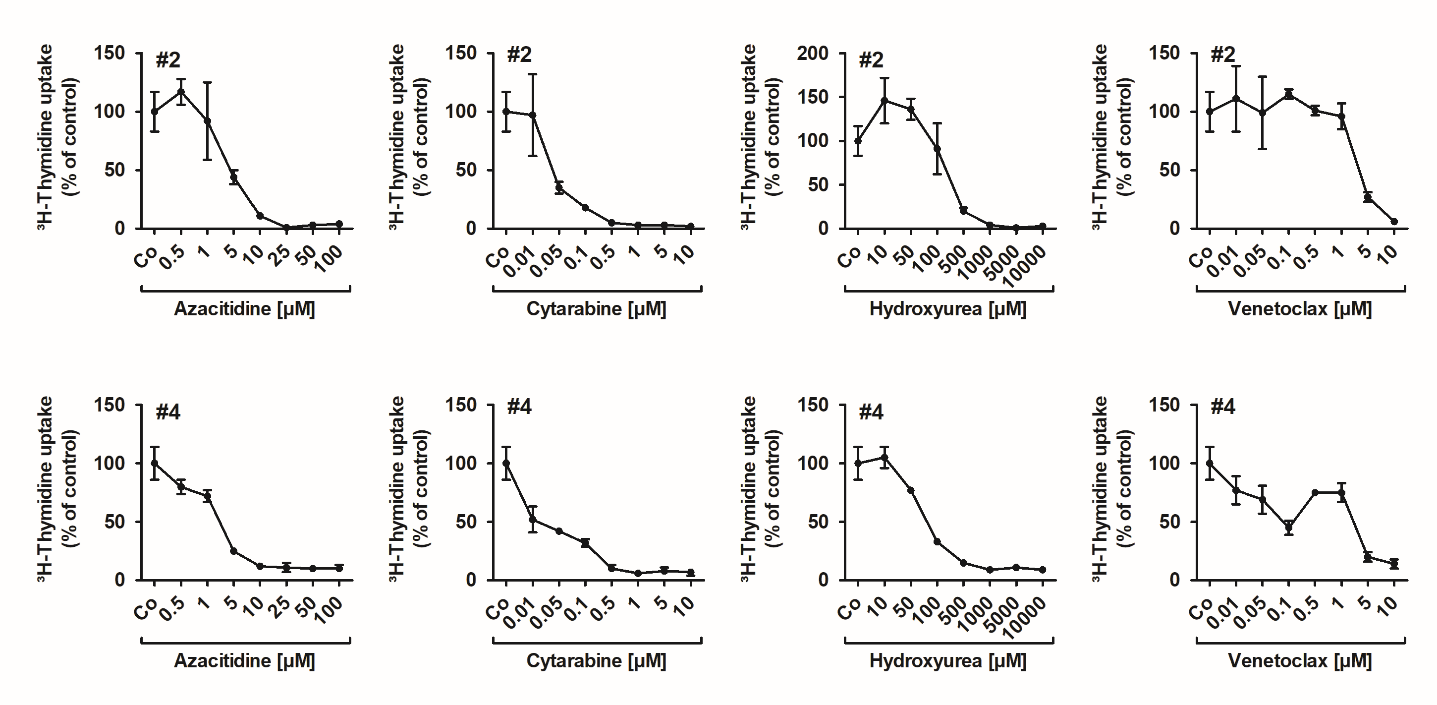
**

**Supplemental Figure S4**

**Effects of targeted drugs on proliferation of primary neoplastic cells**

Primary neoplastic cells obtained from patients #2 (upper images) and #4 (lower images) were incubated in control medium (Co) or in medium containing various concentrations of azacitidine, cytarabine, hydroxyurea, or venetoclax (as indicated) at 37°C for 48 hours. Proliferation was determined by measuring ^3^H-thymidine uptake. Results are expressed as percent of control and represent the mean±SD from triplicates. Numbers of patients (#) refer to the identification numbers shown in Table 1.


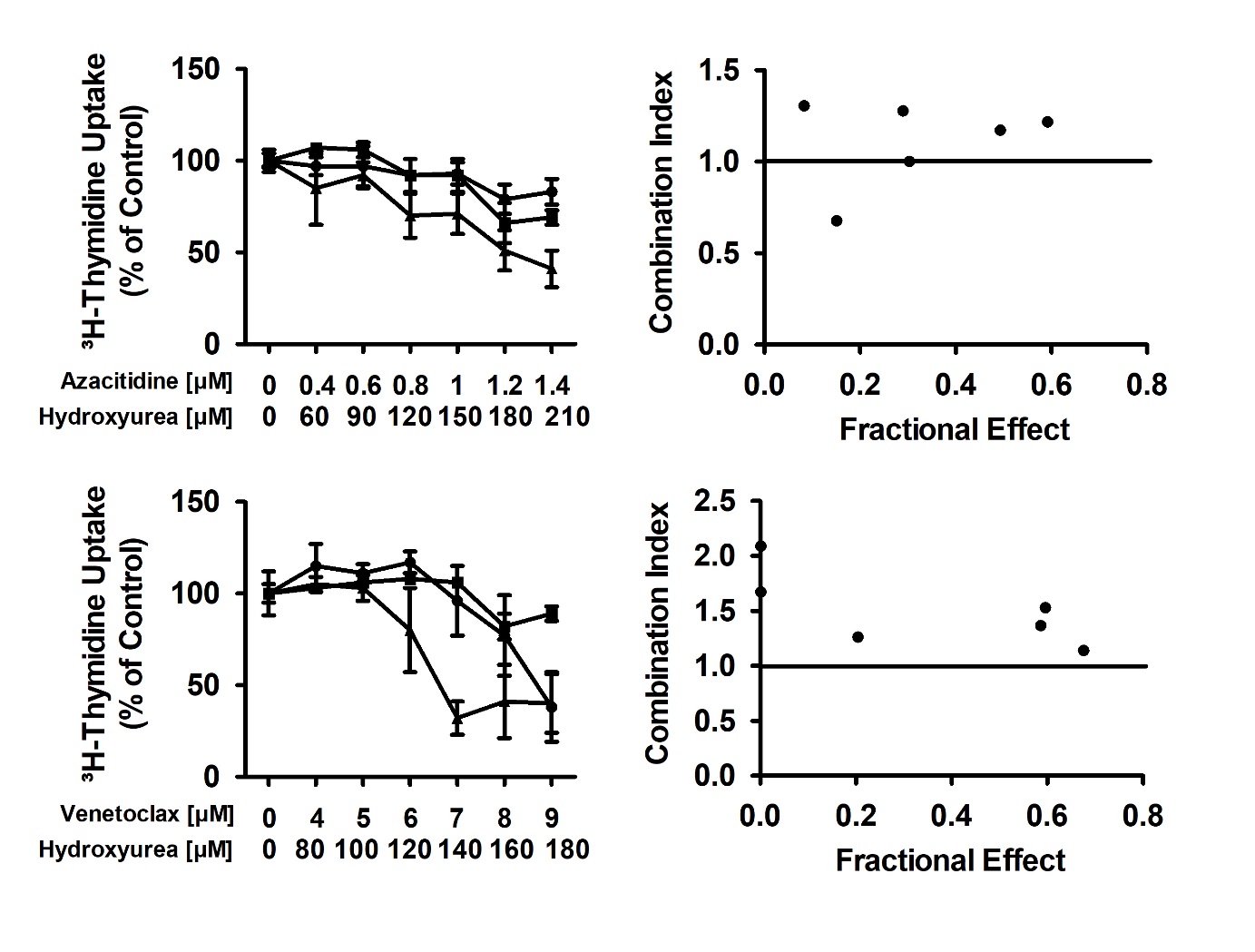


**Supplemental Figure S5**

**Combined drug effect on proliferation of KU812 cells**

KU812 cells were incubated in control medium (0), in medium containing various concentrations of venetoclax, or azacitidine, or hydroxyurea, or combinations of these drugs as indicated, at fixed ratio of drug concentration at 37°C for 48 hours. After incubation, uptake of ^3^H-thymidine was measured. Results are expressed as percent of control and represent the mean±SD of triplicates (left panels). In the right panels, combination index (CI) values, calculated from fractional effects by Calcusyn software, are shown. A CI value of 1 indicates an additive effect and CI values below 1 are indicative of synergistic drug effects.
